# Integrated Multi-Omics Elucidates the Therapeutic Potential and Mechanisms of Andrographolide in Colorectal Cancer

**DOI:** 10.3390/cimb48080783

**Published:** 2026-07-31

**Authors:** Haocheng Guan, Yongchao Li, Menglong Xu, Wenqiang Sun, Tinghui Wu, Shuwei Li

**Affiliations:** 1 The State Key Laboratory Incubation Base for Conservation and Utilization of Bio-Resource in the Tarim Basin of the Xinjiang Production and Construction Corps, Department of Biochemistry and Molecular Biology, College of Life Science and Technology, Tarim University, Alar 843300, China; ghc13703476155@163.com (H.G.); 18850569602@163.com (Y.L.); xumenglong1989@163.com (M.X.); swq9577@163.com (W.S.); 2 School of Medical Technology, Xinjiang Hetian College, Hetian 848000, China

**Keywords:** colorectal cancer, andrographolide, cell cycle arrest, apoptosis, multi-omics

## Abstract

Colorectal cancer (CRC) remains a major clinical challenge owing to its high mortality and limited therapeutic options, highlighting the urgent need for novel anticancer agents. Andrographolide (AGL), the principal bioactive compound isolated from the medicinal herb *Andrographis paniculata*, has demonstrated promising anticancer activity across multiple malignancies; however, its effects on CRC and the underlying molecular mechanisms remain poorly elucidated. Here, we systematically evaluated the anti-CRC potential of AGL using both in vitro and in vivo models. AGL significantly inhibited the proliferation of HT-29 and HCT-116 CRC cell lines in a dose- and time-dependent manner, induced G1-phase cell cycle arrest, suppressed cell migration, and promoted apoptosis and necrosis as determined by CCK-8 assays, flow cytometry, wound-healing assays, and YO-PRO-1/propidium iodide dual staining. Integrated transcriptomic and proteomic analyses of AGL-treated HT-29 cells revealed extensive gene and protein alterations enriched in pathways governing cell cycle control, apoptosis, and cancer-related signaling, and Western blotting confirmed the upregulation of pro-apoptotic proteins and downregulation of anti-apoptotic proteins. Importantly, AGL also significantly suppressed tumor growth in an HT-29 subcutaneous xenograft mouse model. Collectively, these findings demonstrate that AGL exerts potent anti-CRC activity primarily through the induction of G1-phase arrest and apoptosis, supporting its potential as a promising natural lead compound for the development of novel CRC therapeutics.

## 1. Introduction

Colorectal cancer (CRC) is one of the leading causes of cancer-related morbidity and mortality worldwide [1]. In 2022, an estimated 1,926,118 new cases and 903,859 deaths occurred globally, accounting for 9.3% of all cancer deaths [2]. Among new cancer cases diagnosed in the United States in 2025, CRC represents 8% of diagnoses in men and 7% in women, with an estimated 52,900 deaths [3]. Although the survival rate of CRC has improved over recent decades, the 5-year survival rate for metastatic disease remains low [4]. This high mortality is partly attributable to the heterogeneity of the disease, including diverse molecular subtypes, distinct gene mutations, and a unique tumor microenvironment [5,6]. For decades, systemic chemotherapy has been the mainstay for prolonging patient survival, and the most widely used regimens for CRC include 5-fluorouracil (5-FU), oxaliplatin, and irinotecan. Although these conventional chemotherapeutic agents are effective, their toxicity and the emergence of drug resistance limit their long-term use. Therefore, the identification of novel agents for CRC treatment is of great significance.

Andrographolide (AGL) is a diterpenoid lactone extracted from *Andrographis paniculata* (Burm.f. Wall. ex Nees) [7]. It possesses a range of therapeutic properties, including anti-inflammatory, antioxidant, antibacterial, and anti-hyperglycemic activities [8,9,10,11]. AGL also exhibits antitumor effects against multiple cancers, including lung, breast, cervical, liver, and kidney cancers [12,13,14]. Notably, AGL inhibited tumor growth in H1975 xenograft and Lewis lung carcinoma models, with enhanced efficacy at higher doses; by stimulating CD8+ T-cell infiltration and function, it prolonged survival in mice and enhanced the efficacy of anti-PD-1 monoclonal antibody immunotherapy [15]. Although AGL has demonstrated anticancer activity against various cancers, research on its effect against CRC remains limited. Therefore, the present study aimed to investigate the effects of AGL on CRC cells, with particular emphasis on its influence on apoptosis and cell cycle regulation. An integrated transcriptomic and proteomic approach, combined with an in vivo xenograft model, was employed to provide a comprehensive understanding of the therapeutic potential of AGL in CRC.

## 2. Materials and Methods

### 2.1. Reagents and Cell Culture

AGL (Cat. No. SM5172-100 mg; Beyotime Biotechnology, Shanghai, China) was stored at −20 °C. HT-29 cells (Cat. No. CL-0215; Procell Life Science & Technology, Wuhan, China) were cultured in McCoy’s 5A medium (Cat. No. PM150710; Procell) containing 10% fetal bovine serum (FBS; Cat. No. 164210; Procell) and 1% penicillin-streptomycin (PS; Cat. No. PB180120; Procell). HCT-116 cells (Cat. No. CL-0178; Procell) were cultured in DMEM (Cat. No. PM150110; Procell) containing 10% FBS and 1% PS. All cells were maintained at 37 °C in a humidified atmosphere containing 5% CO_2_.The following reagents were also used: Cell Counting Kit-8 (CCK-8; CA1210), bicinchoninic acid (BCA) protein assay kit (PC0020), and phenylmethylsulfonyl fluoride (PMSF; FJP0100), all from Solarbio (Solarbio Science & Technology Co., Ltd. Beijing, China); an apoptosis and necrosis detection kit (C1075S; Beyotime Biotechnology, Shanghai, China); radioimmunoprecipitation assay (RIPA) lysis buffer (PC104-2) and an enhanced-chemiluminescence (ECL) kit (SQ101), both from Epizyme (Epizyme Biomedical Technology Co., Ltd. Shanghai, China).

### 2.2. Cell Proliferation Assay

Cell proliferation was assessed using the Cell Counting Kit-8 (CCK-8) assay. HT-29 and HCT-116 cells (3 × 10^4^ cells/well) were seeded into 96-well plates (CCP-96H; Servicebio Technology Co., Ltd. Wuhan, China) and treated with various concentrations of AGL in their respective culture media for 24 and 48 h. The medium was then discarded, and cells were incubated with 100 μL of medium containing 10% CCK-8 solution (Cat. No. CA1210; Solarbio, Beijing, China) for 1.5–2.0 h. Absorbance was measured at 450 nm using a microplate reader (51119000; Thermo Fisher Scientific Co., Ltd. Waltham, MA, USA). Half-maximal inhibitory concentration (IC_50_) values were calculated using GraphPad Prism 9.5 (GraphPad Software, San Diego, CA, USA).

### 2.3. Cell Cycle Analysis

HT-29 and HCT-116 cells were seeded into 6-well plates at 2 × 10^5^ cells/mL. Cells were treated with AGL at their respective IC_50_ concentrations (28.4 μM for HT-29; 65.3 μM for HCT-116) or with dimethyl sulfoxide (DMSO) vehicle for 48 h. Cells were harvested by trypsinization, fixed in 70% ice-cold ethanol overnight at −20 °C, washed with phosphate-buffered saline (PBS), and stained with 2 μL RNase A (1 mg/mL) and 50 μL propidium iodide (PI; 100 μg/mL) in the dark for 20 min. Cell cycle distribution was analyzed by flow cytometry (BD Biosciences, Franklin Lakes, NJ, USA), and the percentage of cells in each phase (G1, S, and G2/M) was quantified using FlowJo 7.6.1 software (FlowJo LLC, Ashland, OR, USA).

### 2.4. Cell Apoptosis and Necrosis Detection

Cell apoptosis and necrosis were detected using the YO-PRO-1/PI cell apoptosis and necrosis detection kit (Cat. No. C1075S; Beyotime Biotechnology). HT-29 and HCT-116 cells were seeded into 6-well plates and incubated for 24 h at 37 °C in a humidified 5% CO_2_ atmosphere. HT-29 cells were treated with AGL at 28.4 μM (IC_50_), and HCT-116 cells were treated with AGL at 65.3 μM (IC_50_); control cells received vehicle only. After 48 h of incubation, the medium was aspirated, cells were washed once with PBS, and then incubated with 1 mL YO-PRO-1/PI staining solution per well at 37 °C in the dark for 20 min. Fluorescence was observed under a fluorescence microscope. For all downstream 24 h assays, the 24 h IC_50_ concentrations (33.2 µM for HT-29 and 84.8 µM for HCT-116) were used to ensure that the AGL exposure duration in the mechanistic assays matched the time point at which the IC_50_ was determined. The 48 h IC_50_ values (28.4 µM for HT-29 and 65.3 µM for HCT-116) are reported in Section 2.3 to illustrate the time-dependent anti-proliferative activity of AGL

### 2.5. Cell Migration Assay

Migration capacity was evaluated using a wound-healing assay. HT-29 and HCT-116 cells were cultured in 6-well plates until confluent. A linear scratch wound was generated in the cell monolayer using a 200 μL pipette tip (T-200L-C; Servicebio Technology Co., Ltd., Wuhan, China). Detached cells were removed by washing two to three times with PBS, after which cells were cultured in serum-free McCoy’s 5A (HT-29) or DMEM (HCT-116) medium. Wound closure was monitored and photographed at defined time intervals using a light microscope, and the wound area was measured using ImageJ 1.53a software (National Institutes of Health, Bethesda, MD, USA). The migration rate was calculated as: migration rate (%) = (initial wound width − wound width at the end of the experiment)/initial wound width × 100.

### 2.6. Western Blot Analysis

Total protein was extracted from HT-29 and HCT-116 cells, separated by sodium dodecyl sulfate-polyacrylamide gel electrophoresis (SDS-PAGE), and transferred to polyvinylidene fluoride (PVDF) membranes (Millipore, Billerica, MA, USA). Membranes were blocked with blocking buffer (Cat. No. PS108; Epizyme, Shanghai, China) for 15 min at room temperature and then incubated overnight at 4 °C with the following primary antibodies: BCL2-associated X protein (BAX; Cat. No. 50599-2-Ig; 1:1000), caspase-3 (CASP3; Cat. No. 19677-1-AP; 1:1000), β-actin (Cat. No. 81115-1-RR; 1:5000), BH3-interacting domain death agonist (BID; Cat. No. 10988-1-AP; 1:1000), and cytochrome C (CYCS; Cat. No. 10993-1-AP; 1:1000), all from Proteintech (Wuhan, China); and B-cell lymphoma 2 (BCL2; Cat. No. bs-0032R; 1:1000; Bioss, Beijing, China). After three washes with 1× Tris-buffered saline with Tween 20 (TBST), membranes were incubated with horseradish peroxidase (HRP)-conjugated goat anti-rabbit IgG secondary antibody (Cat. No. bs-0295G-HRP; 1:5000; Bioss) for 1 h at room temperature. Protein bands were visualized using an enhanced chemiluminescence (ECL) kit (Cat. No. SQ101; Epizyme) and quantified with ImageJ software. β-actin served as the loading control.

### 2.7. RNA Sequencing and Analysis

HT-29 cells were treated with AGL at the 48-h IC_50_ concentration (28.4 μM) or with DMSO vehicle at 70–80% confluence for 48 h. Four biological replicates were prepared for each group (*n* = 4 for AGL-treated and *n* = 4 for NC-treated), and all replicates were independent biological replicates. Cells were harvested, washed with PBS, centrifuged at 500× *g* for 5 min at 4 °C, and total RNA was extracted using TRIzol reagent (Thermo Fisher Scientific, Waltham, MA, USA). RNA concentration was measured with a Qubit 4.0 fluorometer, and RNA integrity was assessed on a Qsep400 bioanalyzer (BiOptic Inc., New Taipei City, Taiwan); only samples with intact rRNA peaks were used for library construction. Poly(A)^+^ mRNA was enriched from total RNA using Oligo(dT) magnetic beads and fragmented in fragmentation buffer. First-strand cDNA was synthesized with random hexamer primers, followed by second-strand cDNA synthesis with dNTPs and DNA polymerase. Double-stranded cDNA was purified with DNA-binding magnetic beads, end-repaired, A-tailed, and ligated to Illumina sequencing adapters. After size selection, libraries were amplified by PCR to generate the final cDNA libraries. Library concentration was quantified by Qubit, and insert size was verified on a fragment analyzer. Qualified libraries were pooled and sequenced on an Illumina high-throughput sequencing platform using paired-end 150 bp (PE150) chemistry based on sequencing-by-synthesis. A total of 73.55 Gb clean data were generated across the eight libraries, with ≥8 Gb clean data (approximately 60–68 million raw reads and 9.0–10.3 Gb raw bases) per sample and a Q30 base percentage ≥ 95%. Raw reads (FASTQ) were filtered with fastp v0.23.2 by (i) removing adapter-containing reads, (ii) removing paired reads in which either mate contained > 10% ambiguous (N) bases, and (iii) removing paired reads in which >50% of bases had a quality score ≤ Q20. Per-base error-rate and GC-content distributions were inspected for each sample to confirm data quality. Clean reads were aligned to the human reference genome (Ensembl Homo sapiens GRCh38, annotation release 109) using HISAT2 v2.2.1 with default parameters. The overall mapping rate ranged from 97.39% to 97.66%, and the uniquely mapped rate ranged from 92.41% to 94.75% across all samples. Gene-level read counts were obtained with featureCounts v2.0.3. Raw read counts were used as input for DESeq2 v1.38.3. *p*-values were adjusted for multiple testing by the Benjamini–Hochberg (BH) procedure to control the false discovery rate (FDR). Differentially expressed genes (DEGs) were defined as those with |log_2_ fold change| ≥ 1 and adjusted *p*-value (FDR) < 0.05. Principal component analysis (PCA) of the transcriptomic dataset was performed using variance-stabilized transformed (VST) counts generated by DESeq2 after normalization for library size. Pathway enrichment was performed using clusterProfiler v4.6.0 on the Metware Cloud platform (https://cloud.metware.cn, accessed on 15 July 2026).

### 2.8. Proteomic Sequencing and Analysis

HT-29 cells were treated with AGL at the 48 h IC_50_ concentration (28.4 μM) or with DMSO vehicle at 70–80% confluence for 48 h. In parallel with the RNA-seq experiment, proteomic analysis was performed on four independent biological replicates per group (*n* = 4 for AGL-treated and *n* = 4 for NC-treated), and all replicates were independent biological replicates. Cells were harvested, washed three times with ice-cold PBS, centrifuged at 500× *g* for 5 min at 4 °C, and the cell pellets were snap-frozen in liquid nitrogen and stored at −80 °C until use. Sample preparation, LC-MS/MS analysis, and database searching were performed by Metware Biotechnology Co., Ltd. (Wuhan, China). Cell pellets were lysed in ice-cold lysis buffer (8 M urea, 1 mM PMSF, 2 mM EDTA) by ultrasonication on ice for 5 min. Lysates were centrifuged at 15,000× *g* for 10 min at 4 °C, and the supernatants were collected. Protein concentration was determined with a BCA protein assay kit (Beyotime, Shanghai, China). For each sample, 100 μg of total protein was diluted to 200 μL with 8 M urea, reduced with 5 mM DTT at 37 °C for 45 min, and alkylated with 11 mM iodoacetamide in the dark at room temperature for 15 min. After dilution with 800 μL of 25 mM ammonium bicarbonate, samples were digested overnight with 2 μg of trypsin (Promega, V5280) at 37 °C. Digestion was stopped by adjusting the pH to 2–3 with 20% TFA. Peptides were desalted on C18 cartridges (Millipore, Billerica, MA, USA), and peptide concentration was determined with a Pierce™ Quantitative Peptide Assay Kit (Thermo Fisher Scientific). Peptides (200 ng per injection) were separated on a Vanquish Neo UHPLC nano-LC system (Thermo Fisher Scientific) using a trap-and-elute dual-column configuration. The trapping column was a PepMap Neo Trap Cartridge (300 μm × 5 mm, 5 μm), and the analytical column was an Easy-Spray PepMap Neo UHPLC column (150 μm × 15 cm, 2 μm) maintained at 55 °C. Mobile phase A was 0.1% formic acid in water and mobile phase B was 0.1% formic acid in 100% acetonitrile. The flow rate was 2.5 μL/min, with an effective gradient of 6.9 min and a total run time of 8 min. Eluted peptides were analyzed on an Orbitrap Astral high-resolution mass spectrometer (Thermo Scientific) operated in positive-ion DIA mode. Full MS scans were acquired in the Orbitrap at a resolution of 240,000 (at *m*/*z* 200) over an *m*/*z* range of 380–980, with a normalized AGC target of 500% and a maximum injection time of 5 ms. MS2 spectra were acquired in DIA mode with 299 variable isolation windows of 2 Th width, HCD fragmentation at 25% normalized collision energy, a normalized AGC target of 500%, and a maximum injection time of 3 ms. Raw DIA files were processed with DIA-NN v1.8.1 in library-free mode against the Homo sapiens UniProt reference proteome (UP000005640; downloaded 24 February 2025; 83,385 sequences). Deep-learning-based spectral prediction was enabled, and the Match Between Runs (MBR) algorithm was used to generate a project-specific spectral library and re-analyze the DIA data. Both peptide-spectrum-match and protein-level false discovery rates (FDR) were filtered at 1%. Protein quantification was performed with the MaxLFQ algorithm embedded in DIA-NN, followed by sample-wise median normalization. The median number of peptides per protein was ≥1, the proportion of fully tryptic peptides (zero missed cleavages) exceeded 60%, and peptide length distributions ranged from 7 to 20 amino acids, in line with standard quality criteria for DIA-based proteomics. Pearson correlation coefficients between biological replicates within each group were ≥0.85, and the first two principal components of the PCA clearly separated the AGL and NC groups with no overlap, indicating high data quality and reproducibility. Missing values were imputed with the lowest detected value for each protein. Differential abundance between the AGL and NC groups was assessed with Student’s *t*-test on log_2_-transformed MaxLFQ intensities, and fold change (FC) was calculated as the ratio of group means (AGL/NC). Differentially expressed proteins (DEPs) were defined as those with FC ≥ 1.5 or FC ≤ 0.6667 and *p* < 0.05. *p*-values were adjusted for multiple testing using the Benjamini–Hochberg (BH) procedure to control the FDR. Functional annotation of all identified proteins was performed against the GO, KEGG, KOG, and InterPro databases. Subcellular localization was predicted with WoLF PSORT, and signal peptides were predicted with SignalP v5.0b. For each comparison, GO, KEGG, and KOG enrichment analyses of DEPs were performed with clusterProfiler v4.4.4 using a hypergeometric test, and pathways with *p* < 0.05 were considered significantly enriched. Protein–protein interaction (PPI) networks were constructed against the STRING database using DIAMOND blastx v2.0.9 (E-value ≤ 1 × 10^−5^), and interactions with a combined score > 400 were visualized in Cytoscape v3.9.1. Weighted protein co-expression network analysis (WPCNA) was performed with the WGCNA R package v1.69, with the soft-threshold power selected to achieve a scale-free topology fit index (R^2^) ≥ 0.85.

### 2.9. Animal Experiments

Six-week-old male BALB/c nude mice (19–21 g) were purchased from Sipeifu Biotechnology Co., Ltd. (Beijing, China) and used to establish subcutaneous xenograft models. A total of 5 × 106 HT-29 cells were injected subcutaneously into the right axilla. When tumors reached approximately 50 mm^3^, mice were randomized into two groups (*n* = 4 per group): AGL (50 mg/kg) and control (PBS with 5% DMSO). AGL (50 mg/kg in 200 μL vehicle) was administered daily by oral gavage, and control mice received vehicle (200 μL) daily. Tumor size was measured with calipers, and volume was calculated as: volume = (length × width2)/2. At the study endpoint, mice were euthanized, and tumors were excised, weighed, measured, and photographed. Only one sex was used in this exploratory xenograft experiment to maintain consistency of the study design; however, this does not eliminate biological variability, and future studies including both male and female animals will be necessary to assess potential sex-dependent differences in AGL response.

### 2.10. Statistical Analysis

Data are presented as the mean ± standard deviation (SD) from at least three independent experiments. Statistical analysis was performed using GraphPad Prism 9.5 (GraphPad Software). Comparisons between two groups were performed using the Student’s *t*-test, and comparisons among three or more groups were performed using one-way analysis of variance (ANOVA). *p* < 0.05 was considered statistically significant.

## 3. Results

### 3.1. AGL Inhibits the In Vitro Proliferation of HCT-116 and HT-29 Cells

**Figure 1:**
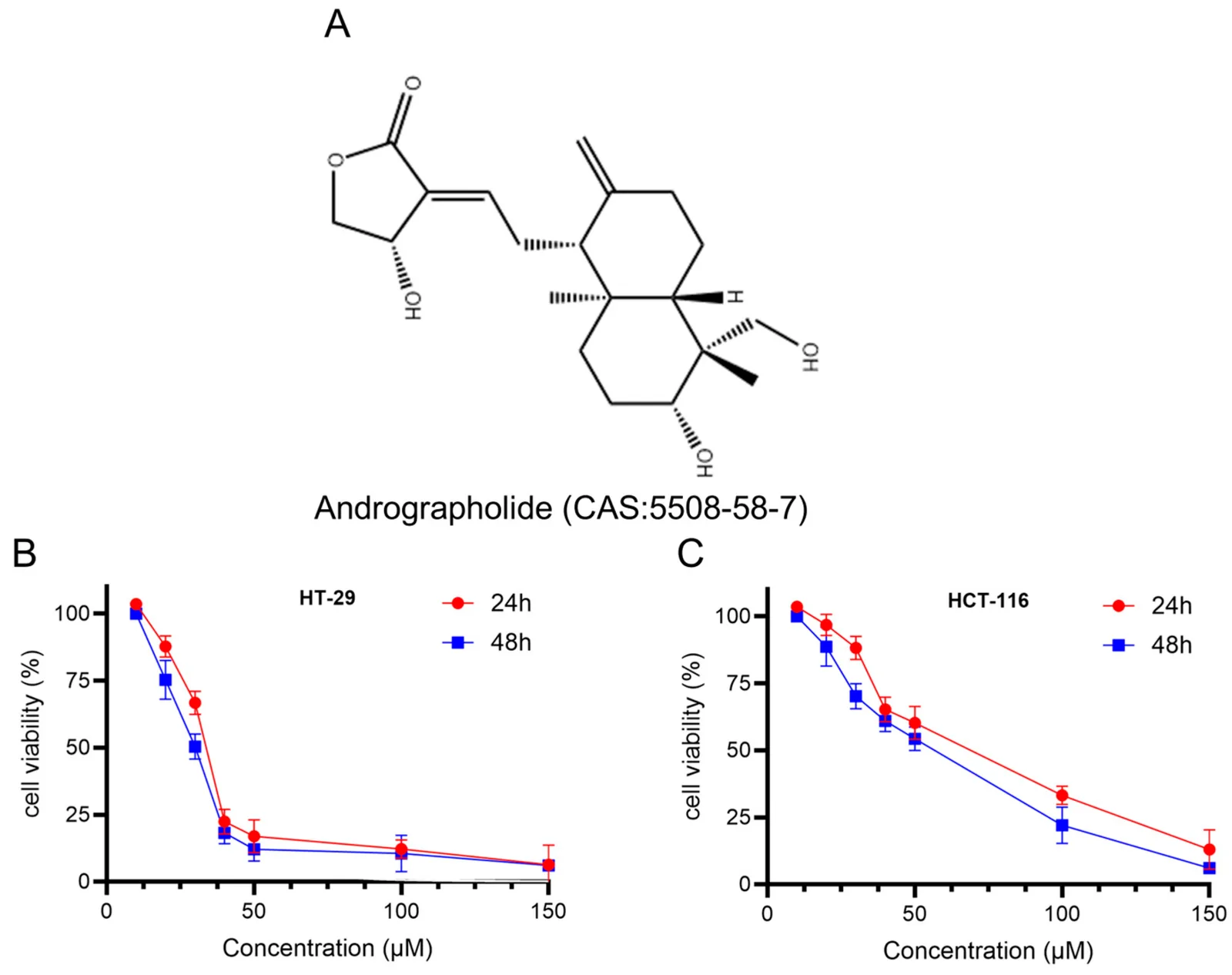
Anti-proliferative activity of AGL against HT-29 and HCT-116 cells. Cell viability after 24 or 48 h of AGL treatment was measured by CCK-8 assay. (**A**) Chemical structure of AGL (CAS: 5508-58-7). (**B**) Dose–response curves showing the effect of AGL on HT-29 cell viability at 24 h (red circles) and 48 h (blue squares). (**C**) Dose–response curves showing the effect of AGL on HCT-116 cell viability at 24 h (red circles) and 48 h (blue squares). Data are presented as the mean ± SD. AGL, andrographolide; CCK-8, Cell Counting Kit-8; SD, standard deviation.

The anti-proliferative effects of AGL on the human CRC cell lines HT-29 and HCT-116 were assessed using CCK-8 assays. Cells were treated with graded concentrations of AGL for 24 and 48 h, and a dose- and time-dependent decrease in viable cell number was observed. At 24 h, the IC_50_ values were 33.2 μM for HT-29 cells and 84.8 μM for HCT-116 cells. At 48 h, the IC_50_ values decreased to 28.4 μM for HT-29 and 65.3 μM for HCT-116 (Figure 1B,C). These data demonstrate that AGL effectively inhibits the proliferation of both HT-29 and HCT-116 cells in vitro.

### 3.2. AGL Induces G1-Phase Cell Cycle Arrest in HCT-116 and HT-29 Cells

**Figure 2:**
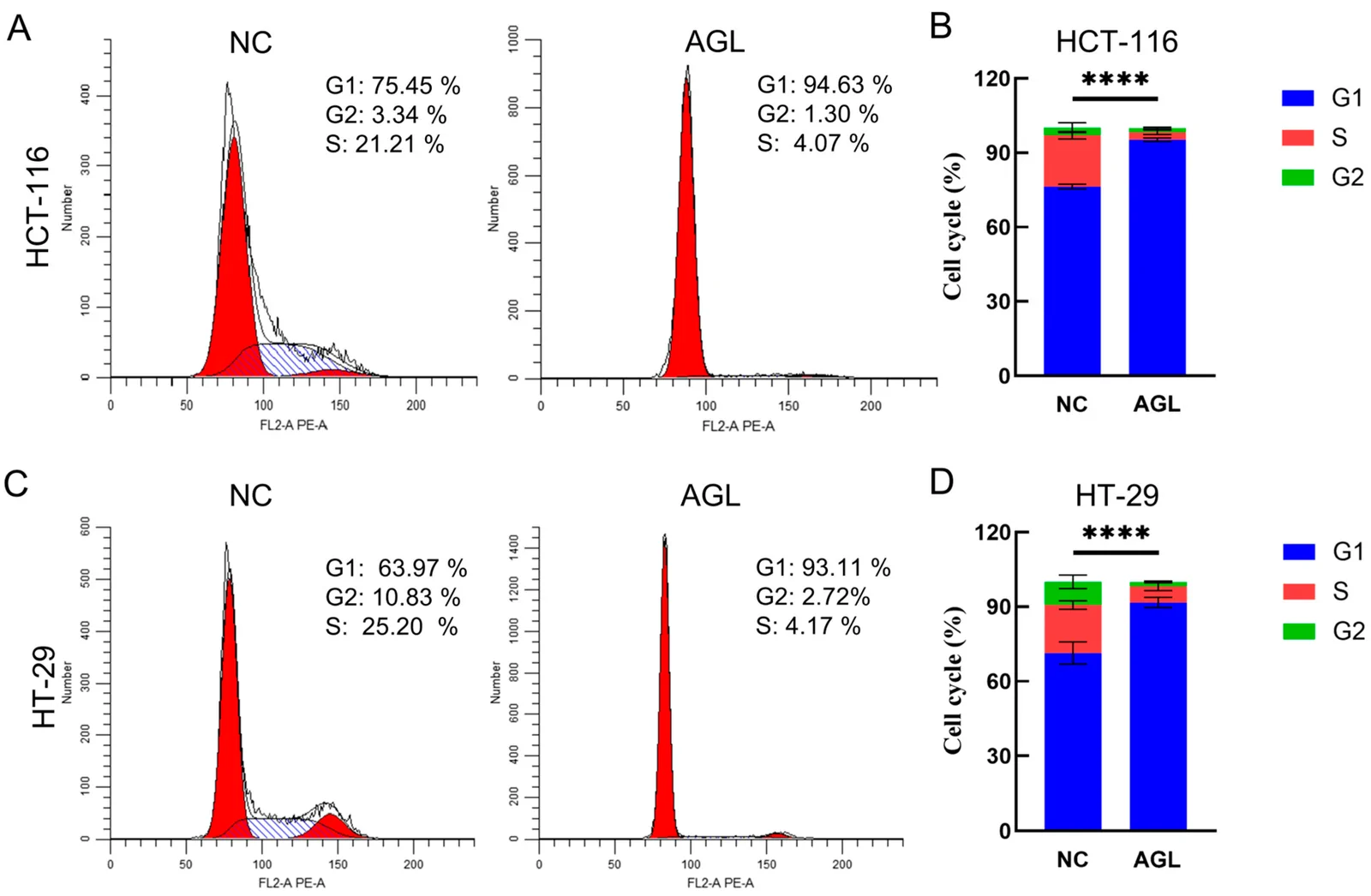
AGL induces G1-phase arrest in HCT-116 and HT-29 cells. Cell cycle status was assessed by flow cytometry after 48 h of AGL exposure, with three independent biological replicates (*n* = 3) (**A**) Representative histograms of the cell cycle distribution of HCT-116 cells (control and AGL-treated). (**B**) Percentage of HCT-116 cells in the G1, S, and G2 phases. (**C**) Representative histograms of the cell cycle distribution of HT-29 cells (control and AGL-treated). (**D**) Percentage of HT-29 cells in the G1, S, and G2 phases. Data are presented as the mean ± SD. ****, *p* < 0.0001 vs. control. AGL, andrographolide; SD, standard deviation.

Flow cytometry analysis revealed that AGL treatment induced G1-phase cell cycle arrest in both HCT-116 and HT-29 cells (Figure 2). In HCT-116 cells, the proportion of cells in G1 phase increased significantly from 75.4% to 94.6% (*p* < 0.0001), accompanied by a decrease in the S-phase population (from 21.2% to 4.1%) and the G2-phase population (from 3.3% to 1.3%) (Figure 2A,B). HT 29 cells displayed a comparable response: the G1 population expanded significantly from 63.9% to 93.1% (*p* < 0.0001), whereas the S phase decreased from 25.2% to 4.2% and the G2 phase from 10.8% to 2.8% after AGL treatment (Figure 2C,D).

### 3.3. AGL Inhibits the Migration of CRC Cells

**Figure 3:**
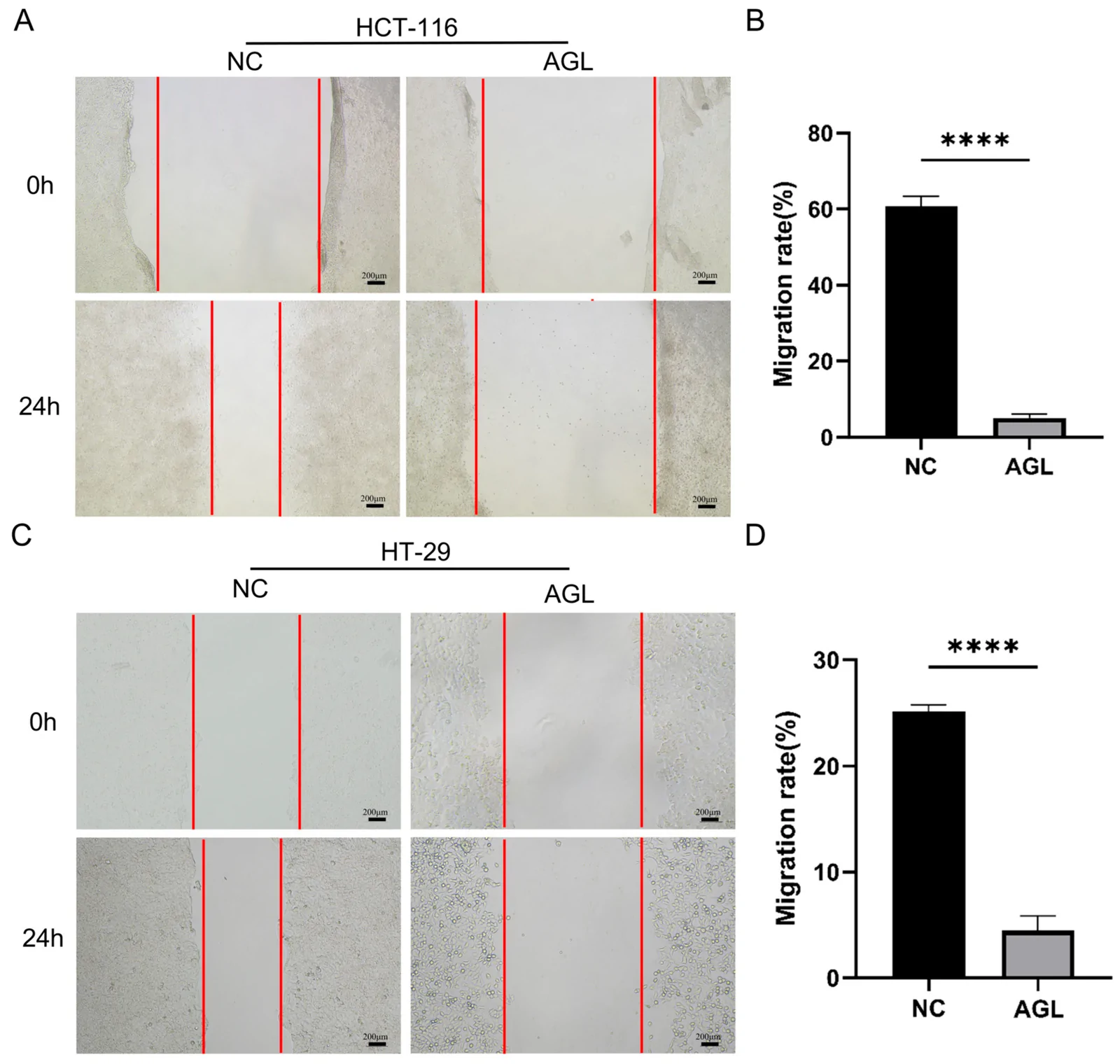
The anti-migratory effects of AGL on HCT-116 and HT-29 cells were evaluated using a wound-healing assay, with three independent biological replicates (*n* = 3) (**A**) Representative images of wound closure over 24 h in HCT-116 cells treated with vehicle (NC) or AGL (0 and 24 h). (**B**) Migration rate (%) of HCT-116 cells in the control and AGL-treated groups. (**C**) Representative images of wound closure in HT-29 cells at 0 and 24 h (NC vs. AGL). (**D**) Migration rate (%) of HT-29 cells exposed to NC or AGL. Data are presented as the mean ± SD. ****, *p* < 0.0001 vs. NC. AGL, andrographolide; NC, negative control; SD, standard deviation.

Wound-healing assays were performed to evaluate the effect of AGL on CRC cell migration. After 24 h, the migration rate of HCT-116 cells decreased significantly from 60.6% in the control group to 5.09% in the AGL-treated group (*p* < 0.001; Figure 3A,B). HT-29 cells showed a comparable reduction, with the migration rate decreasing from 22.54% in the control group to 2.49% in the AGL-treated group over the same period (*p* < 0.001; Figure 3C,D). These results indicate that AGL markedly suppressed wound closure in CRC cells under the tested conditions, suggesting an inhibitory effect on motility-related behavior.

### 3.4. AGL Promotes Apoptosis and Necrosis in CRC Cells In Vitro

**Figure 4:**
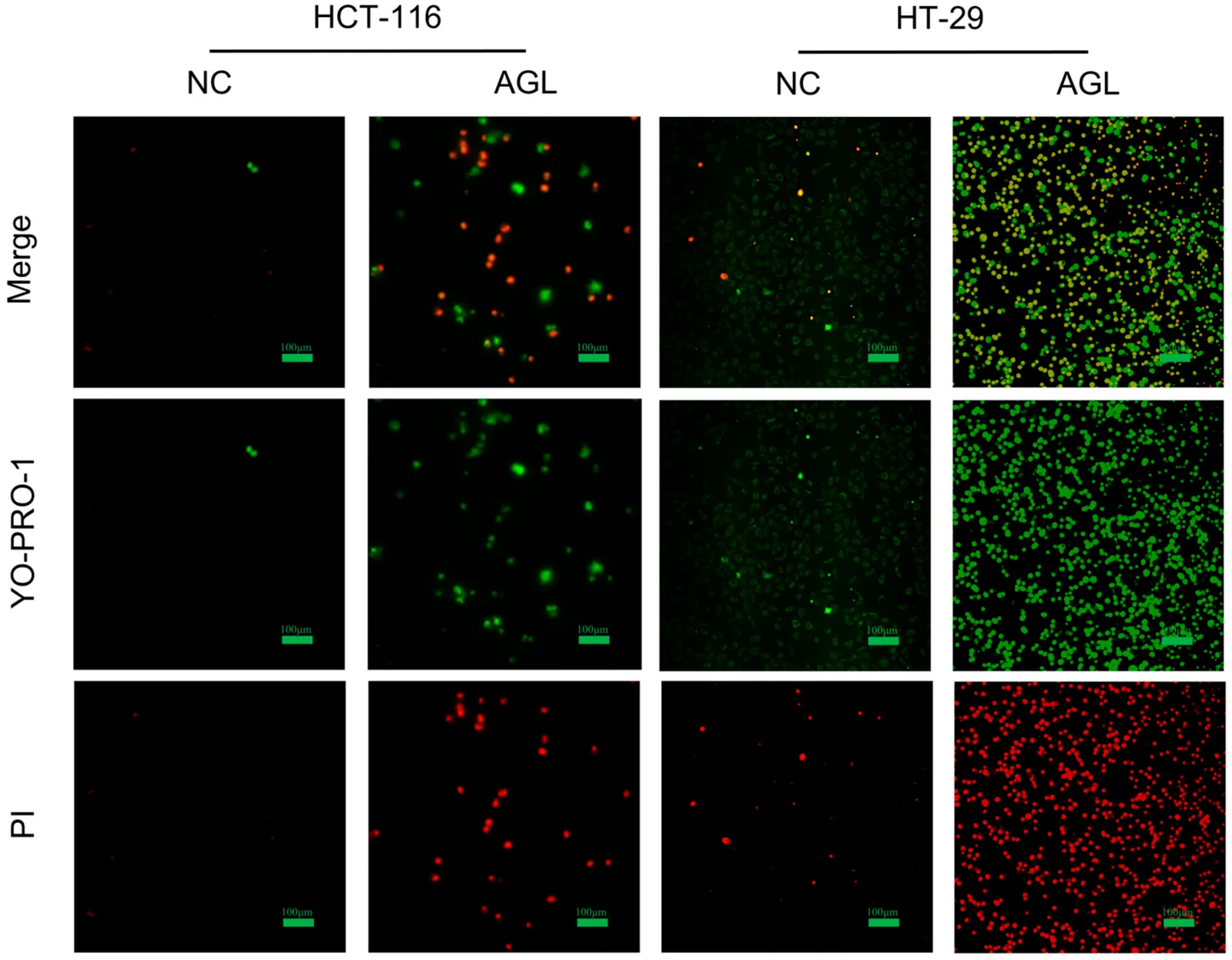
Qualitative assessment of AGL-induced cell death by YO-PRO-1/PI staining in HCT-116 and HT-29 cells, with three independent biological replicates (*n* = 3) for each cell line. Cells were stained with YO-PRO-1 (green) and PI (red) after 48 h of control or AGL treatment to visualize cell death. Representative fluorescence microscopy images of HCT-116 and HT-29 cells under control and treated conditions are shown, displaying merged, YO-PRO-1, and PI signals. Scale bar, 50 μm. AGL, andrographolide; PI, propidium iodide.

To evaluate the ability of AGL to induce apoptosis and necrosis, HCT-116 and HT-29 cells were treated for 48 h and analyzed using the YO-PRO-1/PI kit. Compared with the control group, AGL-treated cells displayed a greater number of cells positive for YO-PRO-1 (indicating early apoptosis) and PI (indicating late apoptosis/necrosis) under microscopic observation (Figure 4), indicating enhanced apoptosis-associated cell death with increased membrane permeability in treated cells.

### 3.5. Integrated Transcriptomic and Proteomic Analysis of AGL-Treated HT-29 Cells

**Figure 5:**
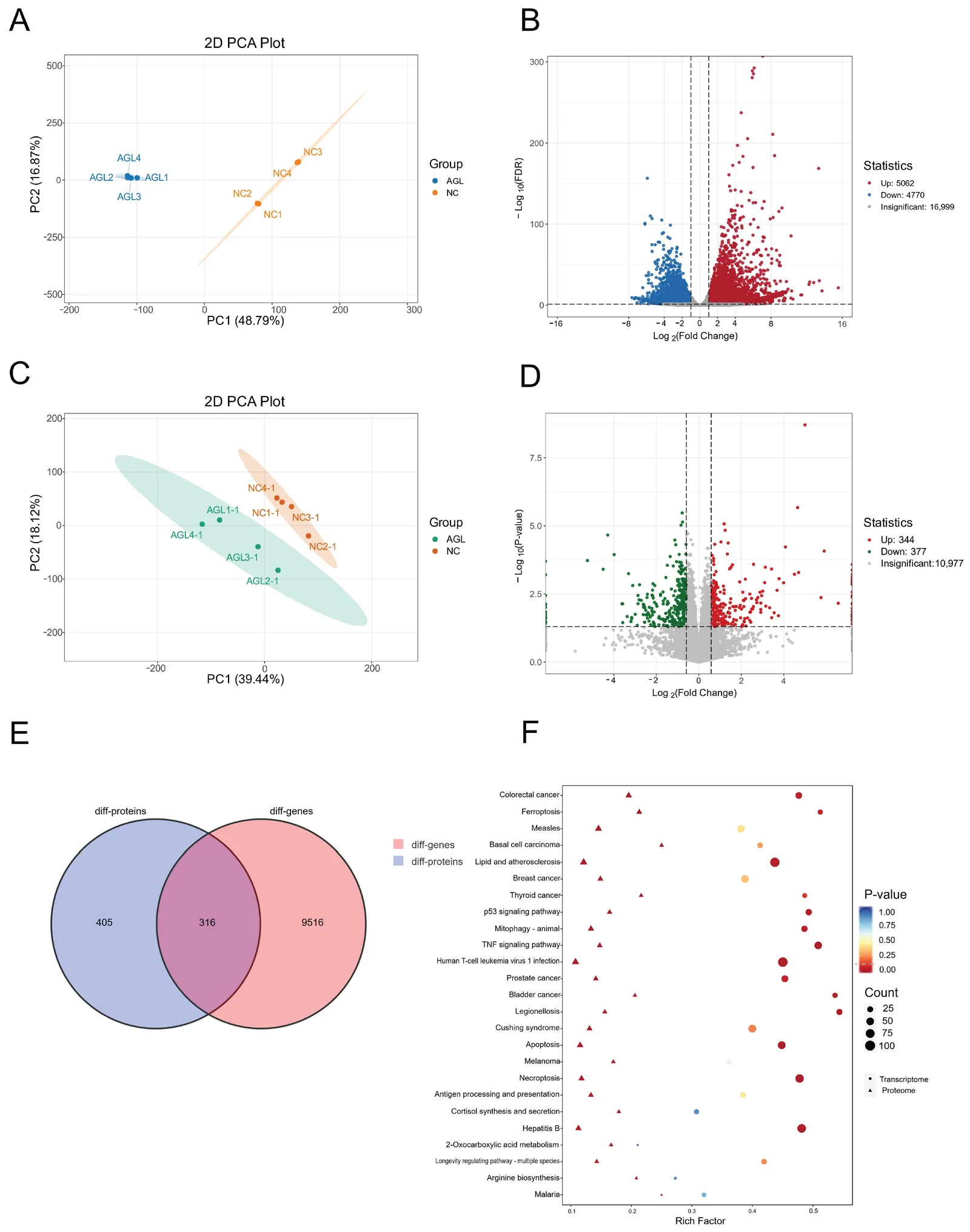
Four independent biological replicates were analyzed for each group (*n* = 4 per group) in both the RNA-seq and proteomic datasets. (**A**) PCA plot of the transcriptomic data generated from variance-stabilized transformed counts, showing separation between the NC and AGL-treated groups. (**B**) Volcano plot of differentially expressed genes in AGL-treated HT-29 cells; red indicates upregulated genes, blue indicates downregulated genes, and gray indicates non-significant genes. Selected top upregulated and downregulated genes are labeled. (**C**) PCA plot of the proteomic data showing separation between the NC and AGL-treated groups. (**D**) Volcano plot of differentially expressed proteins; red indicates upregulated proteins, blue indicates downregulated proteins, and gray indicates non-significant proteins. Selected top upregulated and downregulated proteins are labeled. (**E**) Venn diagram showing the overlap between DEGs and DEPs in AGL-treated HT-29 cells. (**F**) Bubble plot of KEGG pathway enrichment analysis. Bubble size indicates the number of DEGs mapped to each pathway, color indicates the adjusted *p*-value, and the x-axis represents the Rich Factor (Rich Factor = number of DEGs in the pathway/total number of annotated genes in the pathway). AGL, andrographolide; DEG, differentially expressed gene; DEP, differentially expressed protein; KEGG, Kyoto Encyclopedia of Genes and Genomes; NC, negative control; PCA, principal component analysis.

To evaluate the reliability of the multi-omics datasets, quality-control metrics were assessed for both the transcriptomic and proteomic analyses. For the RNA-seq dataset, all eight libraries generated high-quality sequencing output, with at least 8 Gb clean data per sample and Q30 values above 95%. After filtering and alignment, the overall mapping rate ranged from 97.39% to 97.66%, and the uniquely mapped rate ranged from 92.41% to 94.75%, indicating good sequencing quality. Principal component analysis (PCA) based on DESeq2 variance-stabilized transformed counts revealed a discernible shift between the AGL-treated and control groups, although some intra-group variability was observed among the biological replicates. For the proteomic dataset, standard DIA-based quality-control metrics indicated acceptable analytical performance. Most identified peptides were distributed within 7–20 amino acids, and the proportion of fully tryptic peptides with zero missed cleavages exceeded 60%, supporting satisfactory digestion efficiency. Pearson correlation analysis showed good reproducibility among biological replicates within each group (R ≥ 0.85), and PCA demonstrated separation between the AGL and NC groups. Overall, these observations support the suitability of both datasets for downstream integrated analysis. Transcriptomic analysis revealed substantial alterations in the gene-expression profile of HT-29 cells following AGL treatment. PCA based on variance-stabilized transformed counts demonstrated clear separation between the treated and control groups, primarily along PC1 (48.79%) and PC2 (16.87%) (Figure 5A). Volcano plot analysis identified 9832 differentially expressed genes (DEGs), including 5062 upregulated and 4770 downregulated genes (Figure 5B). Proteomic analysis similarly showed marked changes in the protein-expression profile of AGL-treated HT-29 cells. PCA demonstrated distinct separation between the AGL and NC groups along PC1 (39.44%) and PC2 (18.12%) (Figure 5C). Differential expression analysis identified 721 differentially expressed proteins (DEPs), including 344 upregulated and 377 downregulated proteins (Figure 5D). Integrated multi-omics analysis identified 316 molecules that were significantly altered at both the transcript and protein levels (Figure 5E). KEGG pathway enrichment analysis revealed significant enrichment of pathways mainly associated with ferroptosis, the p53 signaling pathway, colorectal cancer, and apoptosis (Figure 5F).

### 3.6. AGL Modulates the Expression of Apoptosis-Related Proteins

**Figure 6:**
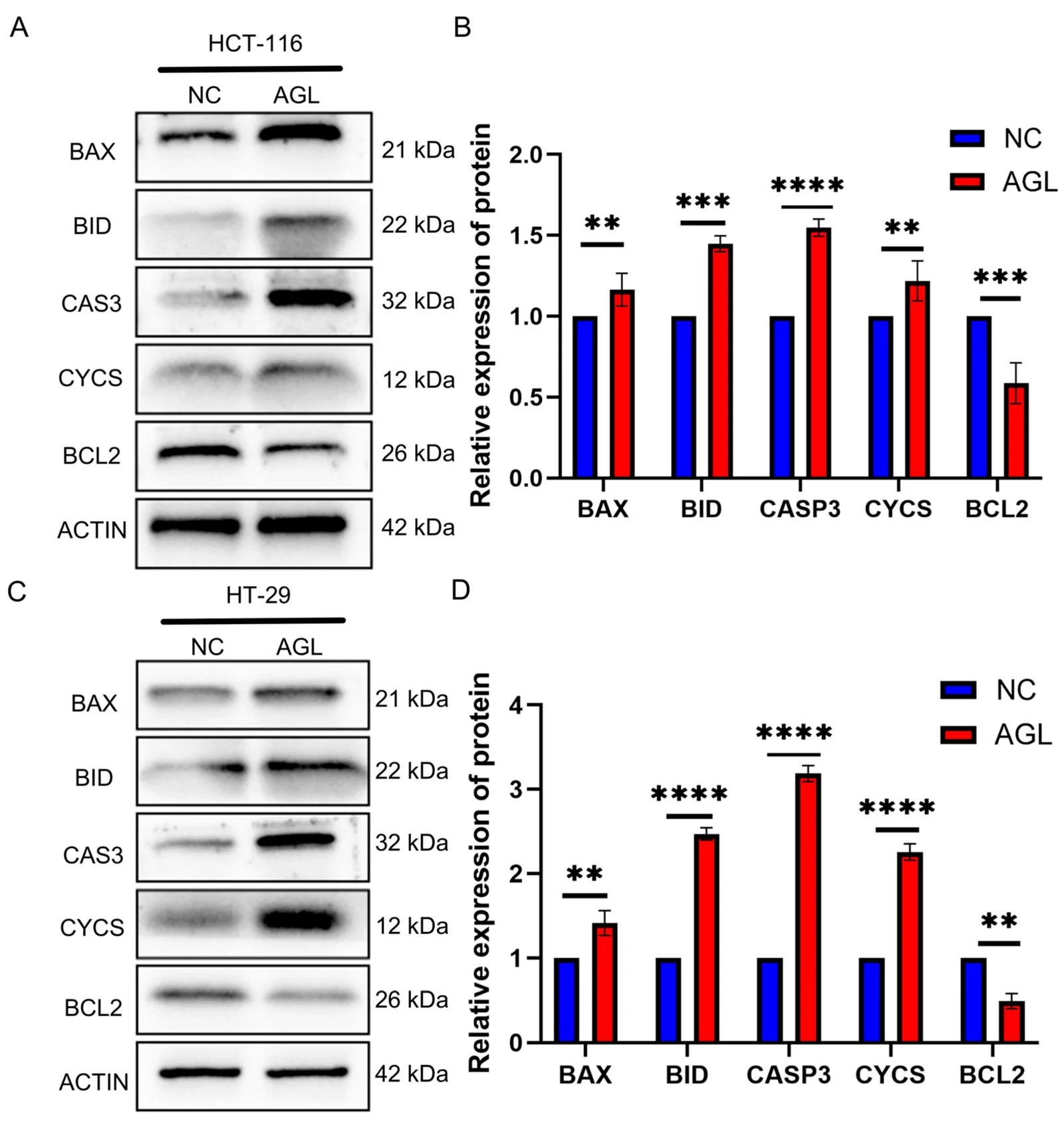
Validation of apoptosis-related protein changes by Western blotting in HT-29 and HCT-116 cells. Cells were treated with AGL for 48 h, and total protein was subjected to Western blot analysis. Three independent biological replicates were analyzed for each cell line (*n* = 3). (**A**) Representative Western blot images of BAX, BID, CASP3, CYCS, BCL2, and β-actin in HCT-116 cells. (**B**) Quantification of protein expression relative to β-actin in HCT-116 cells. (**C**) Representative Western blot images of the same proteins in HT-29 cells. (**D**) Quantification of protein expression relative to β-actin in HT-29 cells. Data are presented as the mean ± SD. ** *p* < 0.01, *** *p* < 0.001, and **** *p* < 0.0001 versus NC. AGL, andrographolide; BAX, BCL2-associated X protein; BCL2, B-cell lymphoma 2; BID, BH3-interacting domain death agonist; CASP3, caspase-3; CYCS, cytochrome c; NC, negative control; SD, standard deviation.

To validate the apoptosis-related alterations identified by integrated transcriptomic and proteomic analyses in HT-29 cells, the expression of key apoptosis-related proteins was examined by Western blotting in both HT-29 and HCT-116 cells. Transcriptomic analysis in HT-29 cells showed that CASP3 (log_2_FC = 2.578, adjusted *p* = 7.76 × 10^−37^), CYCS (log_2_FC = 1.958, adjusted *p* = 1.87 × 10^−7^), and BID (log_2_FC = 0.453, adjusted *p* = 0.0489) were upregulated after AGL treatment. Consistent with these transcriptomic and proteomic findings in HT-29 cells, Western blot analysis showed that AGL treatment for 48 h significantly increased the expression of the pro-apoptotic proteins BAX, CASP3, BID, and CYCS in HT-29 cells, and a comparable induction was also observed in HCT-116 cells (*p* < 0.05; Figure 6A–D). In contrast, the expression of the anti-apoptotic protein BCL2 was significantly reduced in both cell lines following AGL treatment (*p* < 0.05; Figure 6A–D).

### 3.7. AGL Suppresses HT-29 Xenograft Tumor Growth In Vivo

**Figure 7:**
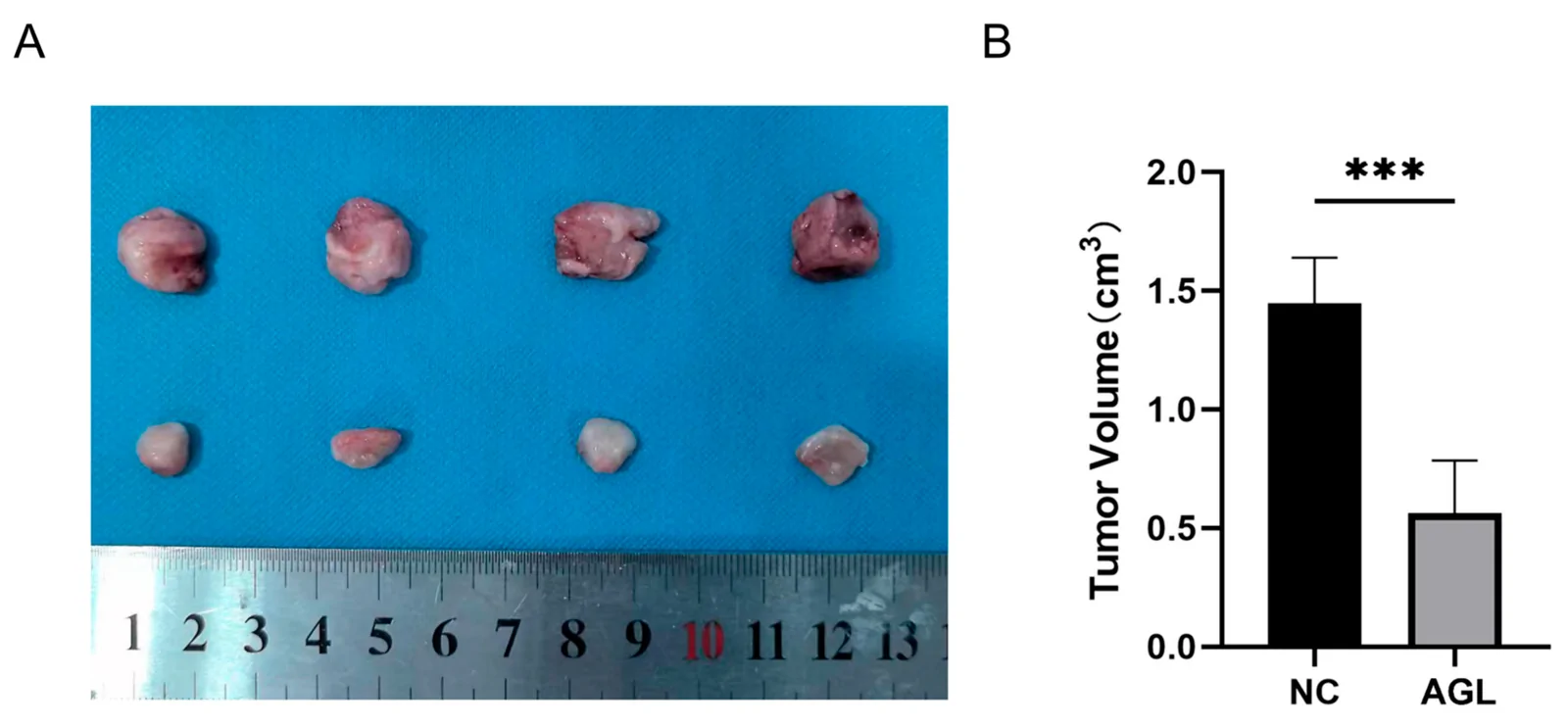
AGL suppresses HT-29 xenograft tumor growth in vivo. (**A**) Representative images of tumors excised from nude mice treated with vehicle control or AGL. (**B**) Tumor volumes measured throughout the study for each group (*n* = 4 per group). The control group received PBS + 5% DMSO by gavage, and the AGL-treated group received 50 mg/kg AGL by gavage. Data are presented as the mean ± SD. ***, *p* < 0.001 vs. control. AGL, andrographolide; DMSO, dimethyl sulfoxide; PBS, phosphate-buffered saline; SD, standard deviation.

The in vivo effect of AGL on HT-29 tumor growth was investigated using a nude mouse xenograft model (Figure 7). Quantitative analysis showed a significant reduction in tumor volume in the AGL-treated group compared with the control group (*p* < 0.01; Figure 7B). This finding was consistent with the macroscopic observation that tumors from AGL-treated mice were smaller than those from control mice (Figure 7A).

## 4. Discussion

The present study demonstrates that AGL exerts potent anti-CRC activity by inducing cell cycle arrest and apoptosis. In vitro, AGL inhibited the proliferation and migration of CRC cells. Integrated transcriptomic and proteomic analyses revealed extensive gene and protein alterations enriched in apoptosis- and cell cycle-related pathways, and these apoptosis-related findings were validated by Western blotting, which showed upregulation of the pro-apoptotic proteins BAX, CASP3, BID, and CYCS and downregulation of the anti-apoptotic protein BCL2. Moreover, AGL significantly suppressed tumor growth in a mouse xenograft model.

Previous studies have confirmed that AGL has broad antitumor potential across different cancer types. Consistent with our observations, AGL has been shown to regulate specific molecular targets in cancer, such as reactivating p53 and inhibiting Mdm-2 in gastric cancer [16] and modulating BAX in breast cancer cells, inhibiting proliferation by downregulating ERα, PI3K, and mTOR [17]. Xuan et al. demonstrated that AGL inhibits proliferation and promotes apoptosis in bladder cancer cells by interfering with NF-κB and PI3K/AKT signaling in vitro and in vivo [18]. Xu et al. reported that AGL inhibits ER-positive breast cancer growth and enhances the efficacy of fulvestrant through the ROS-FOXM1-ER-α axis [19]. Li et al. found that AGL suppresses the growth and metastasis of luminal-like breast cancer by inhibiting the NF-κB/miR-21-5p/PDCD4 signaling pathway [20]. Pasha et al. demonstrated the antitumor activity of AGL in vitro and in vivo, showing significant downregulation of NF-κB and COX-2 and induction of apoptosis through blockade of the PI3K/AKT signaling pathway in cervical cancer [21]. Furthermore, Wang et al. showed that AGL targets DJ-1 to induce ROS accumulation and pancreatic cancer cell death [22]. Building on these findings, the present study demonstrated a similar role for AGL in CRC, with particular emphasis on the regulation of BAX, CASP3, BID, CYCS, and BCL2-mediated G1-phase arrest and apoptosis. Khan et al. showed that AGL inhibited HCT-116 cell proliferation and induced G2/M phase arrest through downregulation of Cyclin B1 and CDK1 [23], and Hong et al. similarly reported AGL-induced cytotoxicity in CRC cells [24]. In line with these previous reports, the present study confirms that AGL exerts anti-proliferative activity in CRC cell lines. Importantly, the present study was not designed to redefine the cytotoxicity profile of AGL, which has been established previously, but rather to use this activity as a defined cellular context in which to investigate the transcriptomic and proteomic landscape of the AGL response. Accordingly, the IC_50_ values and cell-cycle data reported here are used to define the working concentration and the cellular state for the downstream multi-omics and mechanistic analyses, rather than to claim a novel cytotoxic effect. Minor differences in absolute IC_50_ values and in the predominant phase of arrest (G1 in our hands versus G2/M in previous reports) most plausibly reflect methodological variables—cell seeding density, drug exposure duration, assay platform, curve-fitting strategy, and compound source and purity—and indicate that the cell-cycle response to AGL is context-dependent. Together, these observations situate AGL’s anti-CRC activity within an established framework while providing the cellular foundation on which the mechanistic findings of the present study are built. Moreover, AGL has been reported to exert synergistic antitumor effects with cisplatin via ROS-mediated ER stress and STAT3 inhibition. These findings further support the therapeutic value of AGL in colorectal cancer, both as an anticancer agent and as a potential chemosensitizer [24]. Previous studies have indicated that AGL has certain pharmacokinetic limitations, such as limited aqueous solubility and relatively low oral bioavailability, which may influence its in vivo efficiency [7,25]. Formulation and delivery strategies have been reported to improve its cellular exposure and therapeutic potential [14], suggesting that such approaches may further support the future translational application of AGL in CRC.

This study has certain limitations. The findings are based on a limited number of cell lines and lack validation in more clinically relevant animal models, such as orthotopic transplantation models. Although ferroptosis and p53 signaling were among the significantly enriched pathways identified by KEGG analysis, these pathways were not directly validated in the present study and should therefore be considered candidate mechanisms requiring further investigation. Additional studies will be required to further dissect the relative contributions of apoptosis, ferroptosis, and p53-related signaling to the anti-CRC activity of AGL. In addition, the present study did not include parallel evaluation of AGL in normal colonic epithelial cells, which limits the ability to fully assess the tumor-selective activity of AGL. Previous studies have, however, suggested that AGL generally exhibits lower cytotoxicity toward non-malignant cells compared with malignant cell lines [7,9]. In addition, the transcriptomic and proteomic analyses were performed only in HT-29 cells, which limits the generalizability of the omics findings across different CRC models. Future studies should extend these analyses to additional CRC cell lines. Nevertheless, systematic comparison of AGL effects in matched normal and malignant colorectal cells is still needed to better define the therapeutic window of AGL in CRC. Future studies should therefore include a normal colonic epithelial cell line as a control to directly evaluate the selectivity and safety profile of AGL in the context of CRC.

## 5. Conclusions

In conclusion, AGL exhibited significant anti-CRC activity both in vitro and in vivo. AGL effectively inhibited the proliferation and migration of CRC cells, induced G1-phase cell cycle arrest, and promoted apoptosis-related cell death. These findings position AGL as a promising natural compound for the development of novel CRC therapies.

## Data Availability

The data presented in this study are available from the corresponding authors upon reasonable request.

## Abbreviations

The following abbreviations are used in this manuscript:

AGL	andrographolide
CRC	colorectal cancer
CCK-8	Cell Counting Kit-8
PI	propidium iodide
SDS-PAGE	sodium dodecyl sulfate-polyacrylamide gel electrophoresis
PVDF	polyvinylidene fluoride
BAX	BCL2-associated X protein
CASP3	caspase-3
BID	BH3-interacting domain death agonist
CYCS	cytochrome C
BCL2	B-cell lymphoma 2
TBST	Tris-buffered saline with Tween 20
HRP	horseradish peroxidase
ECL	enhanced chemiluminescence
DEGs	differentially expressed genes
DEPs	differentially expressed proteins
PCA	principal component analysis
FDR	false discovery rate
DMSO	dimethyl sulfoxide
FBS	fetal bovine serum
PBS	phosphate-buffered saline
PS	penicillin-streptomycin
SD	standard deviation
ANOVA	analysis of variance
KEGG	Kyoto Encyclopedia of Genes and Genomes
GO	Gene Ontology
IC_50_	half-maximal inhibitory concentration
